# Predictors of adult patient satisfaction with nursing care in public hospitals of Amhara region, Northwest Ethiopia

**DOI:** 10.1186/s12913-019-3898-3

**Published:** 2019-01-21

**Authors:** Ayele Semachew Kasa, Hayleyesus Gedamu

**Affiliations:** Department of Adult Health Nursing, College of Medicine and Health Sciences, Bahir Dar University, Bahir Dar, Ethiopia

**Keywords:** Admitted patient, Nursing care, Interaction model, Amhara region

## Abstract

**Background:**

Nursing care plays a prominent role in determining the overall satisfaction of patients’ hospitalization experience. Studies have shown that satisfaction with nursing care is the best indicator of patients’ satisfaction with healthcare facilities. The aim of the current study was intended to determine the level of satisfaction and identifying factors towards nursing care from the admitted adult patients’ viewpoints.

**Method:**

The study was done from January 01 to February 01/2017 at three public hospitals of Amhara region using an institutional cross-sectional study design. Systematic random sampling technique was employed to recruit 585 sampled study participants. Patient Satisfaction with Nursing Care Scale was utilized to collect the data. Variables which had statistically significant association with the outcome variable (*P* < 0.05) were identified as significant in the multivariable logistic regression analysis.

**Result:**

A total of 563 patients participated. The overall admitted adult patient satisfaction with nursing care was 40.7%. Patients were more satisfied with the provision of health information, affective support, and professional technical control and least satisfied with decisional control which includes allowing patients and their attendants in the involvement of care. Being governmental employee, patients in the age group of 31–40 years and 4–8 patients in a single room were least satisfied with the nursing care whereas ever married, more educated and patients admitted to the surgical ward were more satisfied than their counterparts with nursing care.

**Conclusion:**

The overall level of patient satisfaction in this study was very low in comparison with many studies. This may urge hospital administrators, policymakers and nurses to be more sensitive with patients’ decisional control or sense of autonomy when providing care.

## Background

Patient satisfaction is the patient’s perception of care compared with the care expected. During hospitalization, patient satisfaction embodies a balance between the patient’s perception and expectation of their nursing care [1–3]. Strong emphasis is placed on patient service in an organized manner to comprehend, measure and meet the desires of clients need [4]. Patient satisfaction is related to many social, technical and professional aspects of the care giver as well as the care recipient [5, 6].

The Ethiopian Civil Service Reform is a recent strategy that has been implemented in public institutions to enhance quality of service to the public consumers. This reform has been employed in all hospitals through the country. Based on the reform, understanding client’s’ views is indispensable if any service is to be enhanced or improved. Above all, patient satisfaction is a main indicator of quality care in any healthcare facilities whether in developed nations or developing countries [7] and this quality of care can be assessed by mapping client’s satisfaction with nursing service provided [1, 8–10].

Nursing is an art and science that is directed to keep the individual, family, community and the population as a whole to be healthy by applying the principle of holistic care. Nurses who are working in hospitals are directed to promote comfort, provide a compassionate and respectful care to the client. In addition, nurses are the frontline professional groups involved in the direct patient care giver. In different healthcare systems mainly in hospital settings patients most likely meet up and spend the highest amount of time with nurses during their hospitalization [11]. This will impose nurses to involve in a range of simple to complex activities to maintain quality nursing care so as to enhance patient satisfaction [12]. Nursing care is a key in reducing patient complain and this care is vital not only in maintaining the health status of the admitted patients but also in improving the overall patient satisfaction.

Federal ministry of health in Ethiopia strives to provide quality nursing care service in all health institution [13]. Even though all health institutions are expected to deliver quality nursing care, hospitals are highly expected to provide quality nursing care because hospitals are the only healthcare facilities that provide a comprehensive admission service for clients in Ethiopia. However, there was paucity of studies that showed the level of admitted adult patients’ satisfaction towards nursing care in hospital settings of Amhara region public hospitals. Therefore, this study was intended to determine the level of satisfaction and identifying factors towards nursing care from the admitted adult patients’ viewpoints, Amhara region, Northwest Ethiopia.

## Methods

### Study design and setting

The study was carried out using an institutional cross-sectional study design at three public hospitals of Amhara Region, namely Felegehiwot comprehensive hospital, Debre Tabor hospital, and Finote Selam hospital from January 01/2017 to February 01/2017. Amhara Region is located in northwestern Ethiopia between 9°20′ and 14°20’ North latitude and 36° 20′ and 40° 20′ East longitude. The region has an estimated land area of about 170,000 km^2^ [14]. In this region, there are different zonal and district hospitals that provide admission service to the clients [15]. Selection of the study settings (three hospitals) were based on patient flow and location of hospital in the region in representing different clients view points from different perspectives. Patients who were ≥ 18 years old, conscious, had ≥2 days in hospital stay and who were willing to participate in the study were included.

### Study sample and sampling procedure

Sample size (n) was determined using a single proportion formula using proportion (p) of the satisfied patient in the nursing care of 67% from a study conducted in Addis Ababa [16] level of precision (d) 0.04 at 95% confidence interval (Zα/2). Adding 10% possible nonresponse rate during the actual data collection makes the final sample size 585.

In order to select a representative sample of patients from each hospital, the total number of inpatients in the last six months were obtained from each hospital. Then estimation of the total number of patients that would be admitted during the study period (one month) was done. The obtained sample was proportionally allocated for each hospital. Then systematic random sampling technique was used to select the eligible respondents. Based on this; 292 patients were included from Felegehiwot comprehensive hospital, 176 patients from Debre Tabor hospital and 117 patients were from Finote Selam hospital.

### Data collection

The questionnaire to assess patient’s satisfaction with nursing care was Patient Satisfaction with Nursing Care Scale (PSNCS) which was adapted from a study done in Kuala Lumpur, Malaysia [11]. For the purpose of the current study, the questionnaire had five parts:

- **Part I.** Socio-demographic information.
- **Part II:** Questions related to the patient satisfaction with nursing care. The questionnaire in this section was developed based on Interaction Model of Client Health Behavior which consists of 20 items on a 4-point Likert scale from (1 = strongly disagree, 2 = disagree, 3 = agree, 4 = strongly agree). The questionnaire is divided into four domains: health information, affective support, decisional control and professional-technical competencies [11].
- **Part III:** Questions related to organizational and environmental factors that consist a total of eight items.
- **Part IV:** Questions related to nature of nursing care provided that consists of six items.
- **Part V:** Questions related to type and seriousness of the illness which consists of two items.

### Data collection process and personnel

A total of four personnel were required who were working in a place other than the selected hospitals. Three junior BSc nurses were recruited for data collection and one senior BSc nurse supervised the overall data collection process. All data collectors and the supervisor were given a one-day orientation about the instrument and the data collection process. The questionnaire was prepared in English language and then translated into the local Amharic language and again transcribed into the English language by experts. The pretest was also done on 5% of the total sample size at Debre-Markos hospital. No names or identifying information indicated on the questionnaires. All study participants were assured of confidentiality and anonymity. Then the data were collected through face to face interview.

### Operational definition

Satisfaction was classified into two as satisfied and not satisfied by using demarcation threshold formula (total highest score-total lowest score)/2} + Total lowest score [7].

### Statistical analysis

The data were edited, coded and entered into Epi-Data version 3.1 and exported to IBM SPSS Statistics Version 20 for analysis. Results of the data analysis were presented in the form of descriptive statistics. To check which variables have an association with the dependent variable; bivariate analysis was used primarily. Those variables with *P*-value of ≤0.25 in bivariate analysis were selected to fit into multiple logistic regression model. Finally, variables which had statistically significant association with the outcome variable (*P* < 0.05) were identified as significant with 95% confidence interval (CI). Then the result was summarized and presented by tables, charts, and graphs.

## Results

### Socio-demographic characteristics of study participants

From the total 585 sampled study participants 563 were successfully interviewed yielding a response rate of 96.2%. From the 563 interviewed study participants 329(58.4%) were male. Two hundred twenty-seven (40.6%) of the study participants were found below 30 years of age. Regarding their religion 89.4% were Orthodox Christian Religion follower, 28.6% were single in their marital status, 41.7% were illiterate, 21.0% were a house wife, and 59.9% were residing in the rural part of the region. Two hundred eight-two (50.1%) of study participants were interviewed from Felegehiwot comprehensive hospital whereas 112 (19.9%) were from Finote Selam hospital. Majority of study participants 258 (45.8%) were interviewed from medical ward of all hospitals. Among the interviewed study participants; 120 (21.3%) had history of previous hospital admission in the last 1 year. One hundred seventy (30.2%) got the service free of charge (Table 1).

**Table 1:** Socio-demographic and related characteristics of study participants in public hospitals (*n* = 563) of Amhara Region, 2017

Socio-demographic characteristics of the respondents	N	%
Sex of the respondent		
Male	329	58.4
Female	234	41.6
Age category		
<= 30 years	227	40.6
31–40 years	155	27.6
41–50 years	68	12.1
51–60 years	39	6.9
> 60 years	72	12.8
Religion		
Orthodox	503	89.4
Muslim	47	8.3
Other^a^	13	2.3
Marital Status		
Single	161	28.6
Ever Married^c^	402	71.4
Educational Status		
Illiterate	235	41.7
Read & write only	82	14.6
Elementary (1-8th grade)	104	18.5
Secondary (9 – 12th Grade)	65	11.5
College and above	77	13.7
Occupation		
House wife	118	21.0
Merchant	83	14.7
Governmental employee	66	11.7
Farmer	107	19.0
Student	89	15.8
Others^b^	100	17.7
Residence		
Rural	335	59.9
Urban	226	40.1
Name of hospital		
FelegeHiwote Comprehensive Hospital	282	50.1
Debre Tabor Hospital	169	30.0
FinoteSelam Hospital	112	19.9
Patient admitting unit/ward		
Medical ward	258	45.8
Surgical ward	240	42.6
Orthopedics	65	11.5
Number of patients in a single room		
< 4 patients	84	14.9
4–8 patients	403	71.6
> 8 patients	76	13.5
Hospital stay (in days)		
< 5 days	290	51.5
5 to 10 days	153	27.2
> 10 days	120	21.3
History of hospital admission in the last 1 year		
Yes	120	21.3
No	443	78.7
Payment status of the service		
With payment	393	69.8
Free service	170	30.2
Total	563	100

^a^Catholic & protestant

^b^Daily laborer, retired, Non-Governmental Organization employee, Driver

^c^Ever married = Married, Widowed, divorced

From the total interviewed 563 adult patients; the minimum age of the study participants’ was 18 and 82 years old being the maximum age. The minimum hospital stay for those admitted adult patients in the three hospitals were 2 days and 60 days were recorded as being the maximum days of hospital stay (Table 2).

**Table 2:** Descriptive statistics result of study participants in public hospitals (*n* = 563) of Amhara Region, 2017

Descriptive statistics	Mean	Min.	Max.
Patient’s age (in years)	38.15	18	82
Average monthly income (in Ethiopian birr)	1901.55	150	8000
Hospital stay (in days)	8.89	2	60

1 Ethiopian Birr = 0.037 USD

### Satisfaction and subscale mean score

The overall mean satisfaction percentage score of admitted adult patients was 67.49 (SD = 14.88) with 28.33 minimum and 100 maximum percentage score (Table 3).

**Table 3:** Percentage mean scores for Adult Patient Satisfaction with Nursing Care in public hospitals (*n* = 563) of Amhara Region, 2017

Sub- scale category	Mean	Std. Deviation	Min	Max
Health Information	68.92	17.72	6.67	100.00
Affective support	68.63	17.79	16.67	100.00
Decisional control	60.45	17.20	0.00	100.00
Professional Technical control	68.68	15.76	27.78	100.00
Overall patient Satisfaction	67.49	14.88	28.33	100.00

### Level of satisfaction of patients with nursing care

**Fig. 1:**
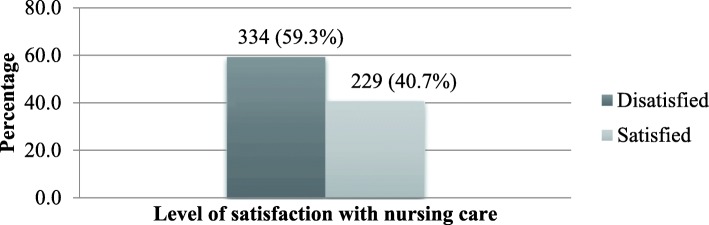
Level of admitted adult patient satisfaction with nursing care in public hospitals (*n* = 563) of Amhara Region, 2017

Two hundred twenty nine (40.7%) of the study participants were satisfied with the care provided by nurses (Fig. 1).

### Percentage description of items in measuring admitted adult patient satisfaction

Three hundred twenty two (57.2%) of the admitted adult patients agreed that nurses explain nursing procedures clearly to them before they perform it. Three hundred fifty seven (63.4%) study participants agreed with the item in which “I receive useful information for the importance of body positioning”. Regarding affective support subscale; 303 (53.8%) disagreed with the item in which nurses use physical touch in supporting them. From the decisional control subscale; 337 (59.9%) of study participants agreed that nurses involve them in hospital care. On the other hand, on professional-technical competency sub-scale; 108 (19.2%) and 102 (18.1%) of study participants disagreed that nurses render nursing care without delay and nurses ask their permission before performing any nursing procedure respectively (Table 4).

**Table 4:** Percentage distribution of items for satisfaction with nursing care in admitted Adult Patients of public hospitals of Amhara Region, 2017

Sub-scale category	Items	Strongly Disagree		Disagree		Agree		Strongly Agree	
		n	(%)	n	(%)	n	(%)	n	(%)
Health information	I receive useful information about my condition from the nurses	3	0.5	65	11.5	351	62.3	144	25.6
	I receive useful information for the importance of body positioning	17	3	71	12.6	357	63.4	118	21
	Nurses explain nursing procedure clearly before they perform it	13	2.3	99	17.6	322	57.2	129	22.9
	Nurses provide me with important information during hospitalization	10	1.8	72	12.8	345	61.3	136	24.2
	Nurses are able to answer my questions in my hospital stay	14	2.5	65	11.5	334	59.3	150	26.6
Affective support	I feel safe when I receive nursing care from nurses	8	1.4	55	9.8	344	61.1	156	27.7
	Nurses use physical touch in supporting me	9	1.6	160	28.4	303	53.8	91	16.2
	Nurses treat me with respect	4	0.7	75	13.3	321	57	163	29
	Nurses are caring	13	2.3	69	12.3	315	56	166	29.5
	Nurses smile whenever they approach me	21	3.7	91	16.2	322	57.2	129	22.9
	Nurses give encouragement to me	4	0.7	79	14	339	60.2	141	25
Decisional control	I can make my own decision when being cared by nurses	10	1.8	149	26.5	330	58.6	74	13.1
	Nurses involve me in hospital care	11	2	159	28.2	337	59.9	56	9.9
	Nurses involve my family/attendant in hospital care	8	1.4	155	27.5	324	57.5	76	13.5
Professional-technical competencies	Nurses are skillful in performing nursing procedures	6	1.1	36	6.4	368	65.4	153	27.2
	Nurses deliver care competently	2	0.4	52	9.2	365	64.8	144	25.6
	Nurses render nursing care without delay	5	0.9	108	19.2	316	56.1	134	23.8
	Nurses ask my permission before performing any nursing procedure	4	0.7	102	18.1	347	61.6	110	19.5
	Nurses are professional when rendering nursing service	13	2.3	56	9.9	349	62	145	25.8
	I have been given privacy from nurses	18	3.2	118	21	340	60.4	87	15.5

### Patient admission by type of problem

**Fig. 2:**
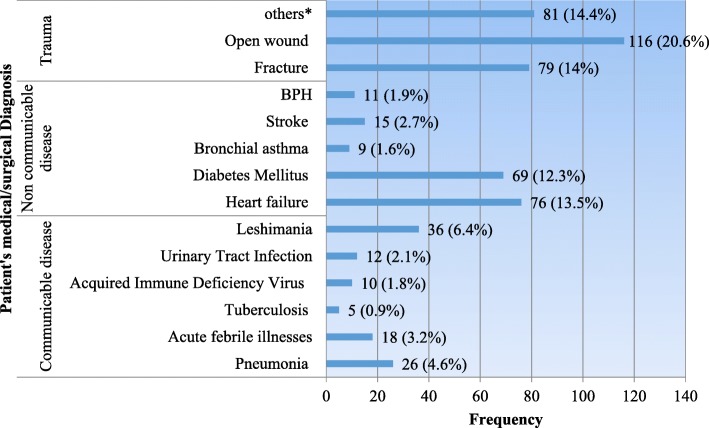
Admitted Adult patient’s medical/surgical diagnosis, Amhara Region public hospitals (*n* = 563), 2017. Others* = anemia, goiter, colelithiasis, appendicitis, Large bowel obstruction, renal calculi, pancytopenia, different tumors. BPH = Benign Prostatic Highpertrophy

Among all the admitted adult patients; 26 (4.6%) were medically diagnosed for pneumonia, 10 (1.8%) diagnosed for HIV/AIDS and 5 (0.9%) diagnosed for tuberculosis. Seventy six (13.5%) diagnosed for heart failure, 69 (12.3%) for diabetes mellitus (Fig. 2).

### Multivariable analysis

Nineteen predictor variables were used in the bivariate analysis and variables having *P* value of < 0.25 were entered into the multivariable logistic regression model. Only six predictor variables significantly associated with the outcome variable. Age of the respondents, marital status, educational status, occupation, patient’s admission ward and a number of patients in a room were variables which had a significant association with admitted adult patient satisfaction with nursing care.

Those respondents who were in the age range of 31 to 40 years were 0.38 times (AOR = 0.38, 95% CI: 0.21, 0.68) less likely to be satisfied than those who were below 30 years of age. Ever married patients were 2.4 times (AOR = 2.42, 95% CI: 1.4, 4.2) more likely to be satisfied than their single counterparts. Regarding educational level those who were 9 to 12th grade completed were almost 2 times (AOR = 2.13, 95% CI: 1.07, 4.26) and those who were college and above were 3.45 times (AOR = 3.45, 95% CI: 1.2, 9.8) more likely to be satisfied than their illiterate counterparts respectively. The governmental employee admitted patients were 0.2 times (AOR = 0.19, 95% CI: 0.05, 0.60) less likely to be satisfied than housewife respondents. Patients who were admitted to surgical ward were 1.6 times (AOR = 1.6, 95% CI: 1.1, 2.51) more likely to be satisfied than patients who were admitted to medical ward. Those patients who were 4 to 8 in a room were 0.33 times (AOR = 0.33, 95% CI: 0.18, 0.6) less likely to be satisfied than patients who were less than 4 in a single room (Table 5).

**Table 5:** Predictors of admitted adult patient’s satisfaction with nursing care, Amhara Region public hospitals (*n* = 563), 2017

		Level of satisfaction		COR (95% CI)	AOR (95% CI)
	Variable	Satisfied	Dissatisfied		
Age of respondents	< 30 years	101 (44.5%)	126 (55.5%)	1	1
	31–40 years	47 (30.3%)	108 (69.7%)	0.54 (0.35,0.83)	0.38 (0.21,0.68)*
	41–50 years	26 (38.2%)	42 (61.8%)	0.77 (0.44,1.34)	
	51–60 years	17 (43.6%)	22 (56.4%)	0.96 (0.48,1.9)	
	> 60 years	38 (52.8%)	34 (47.2%)	1.39 (0.81,2.37)	
Marital status	Single	58 (36.0%)	103 (64.0%)	1	1
	Ever married^a^	171 (42.5%)	231 (57.5%)	1.3 (0.9,1.9)	2.42 (1.4,4.2)*
Educational status	Illiterate	89 (37.9%)	146 (62.1%)	1	1
	Read & write only	32 (39.0%)	50 (61.0%)	1.05 (0.63,1.76)	
	Elementary (1-8th grade)	46 (44.2%)	58 (55.8%)	1.3 (0.81,2.1)	
	Secondary (9 – 12th Grade)	36 (55.4%)	29 (44.6%)	2.0 (1.17,3.5)	2.13 (1.07,4.26)*
	College and above	26 (33.8%)	51 (66.2%)	0.84 (0.48,1.44)	3.45 (1.2,9.8)*
Occupation	House wife	43 (36.4%)	75 (63.6%)	1	1
	Merchant	39 (47.0%)	44 (53.0%)	1.55 (0.87,2.74)	
	Governmental employee	14 (21.2%)	52 (78.8%)	0.47 (0.23,0.94)	0.19 (0.05,0.6)*
	Farmer	46 (43.0%)	61 (57.0%)	1.3 (0.77,2.24)	
	Student	35 (39.3%)	54 (60.7%)	1.13 (0.64,1.99)	
	Others^b^	52 (52.0%)	48 (48.0%)	1.9 (1.1, 3.25)	
Hospital name	Felegehiwote referal hospital	101 (35.8%)	181 (64.2%)	1	1
	Debretabor hospital	82 (48.5%)	87 (51.5%)	1.7 (1.15,2.49)	
	Finoteselam hospital	46 (41.1%)	66 (58.9%)	1.25 (0.8,1.9)	
Ward	Medical	81 (31.4%)	177 (68.6%)	1	1
	Surgical	115 (47.9%)	125 (52.1%)	2 (1.4,2.9)	1.6 (1.1,2.51)*
	Orthopedics	33 (50.8%)	32 (49.2%)	2.25 (1.29,3.9)	
Number of patients in a room	< 4 patients	55 (65.5%)	29 (34.5%)	1	1
	4–8 patients	139(34.5%)	264(65.5%)	0.28 (0.17,0.45)	0.33 (0.18,0.6)*
	> 8 patients	35 (46.1%)	41 (53.9%)	0.45 (0.24,0.85)	
Pt’s file having Nursing process	Yes	203 (44.4%)	254 (55.6%)	1	1
	No	26 (24.5%)	80 (75.5%)	0.41 (0.25,0.65)	
Having > = 2 med/surg diagnosis	Yes	29 (35.8%)	52 (64.2%)	1	1
	No	200 (41.5%)	282 (58.5%)	1.27 (0.78,2.1)	

Ever married^a^ = Married, Widowed, divorced, COR = Crudes Odds Ratio and AOR = Adjusted Odds Ratio

Others^b^ = Daily laborer, retired, Non-Governmental Organization employee, Driver

* significant at *P* < 0.05

## Discussion

Nursing staff are the most numerous professional group and have the greatest contact with patients in comparison with physicians and other healthcare professionals. Therefore, they have significant chance to manipulate patients’ attitudes and behaviors’ in relation to their care, rehabilitation, and recovery process [16, 17]. Patient satisfaction with nursing care is an important indicator of the quality of care provided in hospitals [18–21]. The main objective of this study was to determine the level of adult admitted patients’ satisfaction in nursing care and factors affecting their satisfaction in Amhara region public hospitals.

The proportion of satisfied admitted adult patients with the nursing care in the current study was (40.7%) lower than different studies in Ethiopia. A study done in Dessie Referral Hospital, Jimma University Specialized Hospital, Addis Ababa and in the eastern part of Ethiopia showed that 52.5, 61.9, 67 and 52.75% of admitted adult patients were satisfied with the nursing care respectively [2, 7, 16, 22].

The possible explanation for variations with those studies might be lower sample size, the inclusion of single institution, and the inclusion of obstetrics ward in which most of the time clients in this ward are with higher satisfaction rate comparing with other patients in other wards. In addition data collectors from a similar institution were involved in the data collection process which may increase biased result.

Different studies in different countries revealed variations in the level of patient satisfaction with nursing care. A study done in Lebanon showed that 96.6% [23], Iran 69, 82.8% [1, 3] Kenya 67.8% [4], Malaysia 61.4% [24] and a study done in District Headquarter Hospital Khan revealed 45% [25] of patients were satisfied with the nursing care they received.

The discrepancies in the level of adult patient satisfaction with nursing care in the current study and with studies conducted in abroad might have different explanations. Studies conducted in Kenya and District Headquarter Hospital Dera Ismail Khan hospital were used nonprobability sampling methods, smaller sample size, differences in study populations, and specific tasks to be implemented may raise patient’s satisfaction. Whereas study conducted in Iran consists study populations with specific medical diagnosis, data collected from a single institution and comprising medical services in addition to nursing care which may boost satisfaction level [1, 3]. The possible explanation for variations with a study conducted in Malaysia with the current study might be, the current study includes medical ward which is usually believed to be the commonest ward with high patient dissatisfaction, while the Malaysia study focuses only on patients in orthopedics ward [22]. In addition to the above explanation, there may be better professional expertise and adequate technology to implement nursing and medical services which leads better nursing care practices so that patients will have better satisfaction.

The top aspects of admitted adult patient satisfaction in nursing care in this study were the provision of health information, professional technical competency and affective support respectively whereas patients from decisional control were least satisfied. This finding is in line with a study conducted in Malaysia [11], Jordan [26], Philippine General Hospital [10] Iran [27] except participants had the lowest score in the communication domain and study done in Punjab, India [12].

The current finding also supported with a study conducted in different regions of the world in which patients were less satisfied when nurses did not recognize their opinions during hospital care [28–30].

In the current study patients in the age group of 31–40 years were 0.38 times less likely to be satisfied with nursing care as compared to patients below the age of 30 years of age. This might be due to an increment in age may have the greater expectation with the care provided and also older patients might need support for their activity of daily living from nurses and if their needs were not met in turn this may decrease their satisfaction.

Ever married patients were more satisfied than their single counterparts and patients who were admitted to surgical wards were more satisfied than patients admitted to medical wards. This finding was consistent with a study done in Jimma University Specialized Hospital and Dessie Referral Hospital [2, 22].

Governmental employees were less likely to be satisfied than their housewife counterparts. This finding was consistent with a study done in Northwest Ethiopia [13].

Those patients who were more educated were found to be more satisfied than their less educated/illiterate counterparts. This finding was supported by different studies in which they revealed that patient satisfaction was influenced by the educational level of the respondents [3, 23, 31].

### Strength and limitation of the study

#### Strengths

The current study utilized a valid and standardized instrument (Patient Satisfaction with Nursing Care Scale) and relatively large sample size (*n* = 585) with 3.8% non-response rate was achieved. The finding tried to share-out one of the healthcare system indicator which is nursing care.

#### Limitation

Because of time constraints, the interview was conducted on admitted adult patients. These admitted patients may be afraid to say what they feel about the nursing care because they are still in the ward and they may feel their saying may affect their next nursing care. So, the finding of the current study might be overestimated when we compared to the real patient perceptions.

To avoid such feelings, exit interview using qualitative method will be useful because patients are already left the ward and they will suggest what they feel.

## Conclusion

In comparison with many national and international studies, the current study revealed that admitted adult patients had low level of satisfaction with nursing care. Hence, the current finding will have direct implication towards nurse professionals, hospital administrators (Ward Heads, Matrons, and Hospital Chief Executive Officers) and other concerned Regional and Federal Health Bureaus. Hospital administrators and nurses need to be more concerned with the overall quality of nursing care including patients’ sense of autonomy when providing care.

Nurses may need on site refresher trainings, strengthening morning rounds and establishing nursing quality audit team. By doing this, the nursing quality will be improved and maintained so that patients will receive improved nursing care.

The present study also revealed that as the number of admitted patients in a single room increased there was an increased chance of patients being least satisfied with the care being provided. So, hospital administrators and other concerned government officials shall think on improving the expansion of admission rooms so that patients will access enough rooms with limited number of patients in a room. Moreover, recommendation for additional research would be suggested using through qualitative research method to explore the insight of patients’ satisfaction with nursing care.

## Abbreviations

AIDS: Acquired immune deficiency virus
AOR: Adjusted odds ratio
CI: Confidence interval
COR: Crude odds ratio
HIV: Human immune virus
